# Case report: A rare case of gelatinous pericardial effusion in a COVID-19 positive breast cancer patient

**DOI:** 10.3389/fonc.2026.1743453

**Published:** 2026-03-11

**Authors:** Nouf Alanazi, Sara Abou Al-Saud, Khalid Aljohani, Khalid AlSaleh

**Affiliations:** 1 Cardiac Sciences Department, College of Medicine, King Saud University, Riyadh, Saudi Arabia; 2 Hematology and Oncology Department, College of Medicine, King Saud University, Riyadh, Saudi Arabia

**Keywords:** breast cancer, COVID-19, CVD (cardio vascular disease), gelatinous-fluid, pericardial effusion

## Abstract

**Background:**

Pericardial effusion can occur as a complication of breast cancer and coronavirus disease (COVID-19). However, the specific characteristics of pericardial fluid in patients concurrently experiencing both conditions remain poorly understood.

**Case summary:**

This report describes a 52-year-old woman with a history of hypothyroidism, diabetes mellitus, and breast cancer who developed recurrent pericardial effusion complicated by cardiac tamponade following COVID-19 infection. The patient initially underwent pericardiocentesis and subsequently required pericardiectomy. Notably, the pericardial fluid obtained during pericardiocentesis exhibited a distinctive gelatinous appearance, a feature not previously described in association with breast cancer or COVID-19.

**Conclusion:**

This case highlights the potential for unique interactions between breast cancer and COVID-19, particularly in the development of pericardial effusions with unusual characteristics and a gelatinous appearance. Further studies are warranted to elucidate the underlying mechanisms and clinical implications of this association.

## 1 Introduction

Pericardial effusion is a well-documented complication of COVID-19, particularly in patients with severe disease. While the exact prevalence varies across studies, depending on factors such as COVID-19 severity, lung involvement, age, and the specific population studied, several studies estimate its occurrence at 4-14%. Furthermore, pericardial effusion is associated with higher cardiovascular mortality and worse outcomes (1, 2). Pericardial effusion can occur as a complication of breast cancer, although it is relatively uncommon. In contrast, it has been increasingly recognized as a cardiovascular complication of COVID-19. However, the reported incidence varies widely. The coexistence of breast cancer and COVID-19 may potentially increase the risk of pericardial effusion formation. Pericardial fluid characteristics in malignant pericardial effusions often exhibit a hemorrhagic appearance, high protein content, and may contain malignant cells. Similarly, reports have suggested the exudative nature of the fluids in the setting of COVID-19. While gelatinous fluid has been reported in pleural effusions associated with certain malignancies (3, 4), it has not been reported with breast cancer-related pericardial effusions. To our knowledge, this is the first case report that describes a jelly-like pericardial effusion in a patient with breast cancer and COVID-19 infection.

## 2 Case report

A 52-year-old female presented to the emergency department with chest pain for 5 days before her presentation, associated with fever (at ~39 °C), cough, shortness of breath, and diaphoresis (**Table 1**). The pain was stabbing and aggravated mainly by coughing. She had been previously diagnosed with diabetes mellitus, hypothyroidism, and breast cancer (estrogen receptor-positive, progesterone receptor-positive, and HER-2-Neu positive). The latter was diagnosed in 2015, and neo-adjuvant chemotherapy was initiated in January 2016, including 5-fluorouracil, epidoxorubicin, and cyclophosphamide. The patient was then prescribed docetaxel and trastuzumab therapy in March of 2016. During the course of the therapy, she developed transient left ventricular dysfunction that resolved with goal-directed medical therapy. Her last echocardiography after completing her therapy and a MUGA scan showed an ejection fraction of 56% in 2017. The patient remained in remission until her last visit in September 2020. Her last mammogram at that visit showed benign left breast findings (BI-RADS 2), and routine annual screening mammography was recommended.

**Table 1:** Timeline of the case.

Timeline	Event
On admission	A 52-year-old female admitted with COVID-19 related pneumonia and pericardial effusion complicated with pericardial tamponade.
1st week after admission	Pericardiocentesis revealing a gelatinous pericardial fluid.
3rd week after admission	Recurrent effusion requiring pericardiectomy.
4th week after admission	Diagnosis of breast cancer recurrence with evidence of metastasis requiring systemic therapy.
Follow up	Stable on the current systemic therapy regimen with good quality of life.

### 2.1 History of examination

The patient was conscious and oriented on examination; her vital signs were 36.5 °C, blood pressure 144/81 mmHg, HR 110 bpm, oxygen saturation of 92%, and Body Mass Index of 33.7. Chest examination revealed bilateral wheezing and reduced breath sounds in the right lower lung field with distant S1 and S2 and no murmurs or pericardial rub.

### 2.2 History of investigations

**Figure 1:**
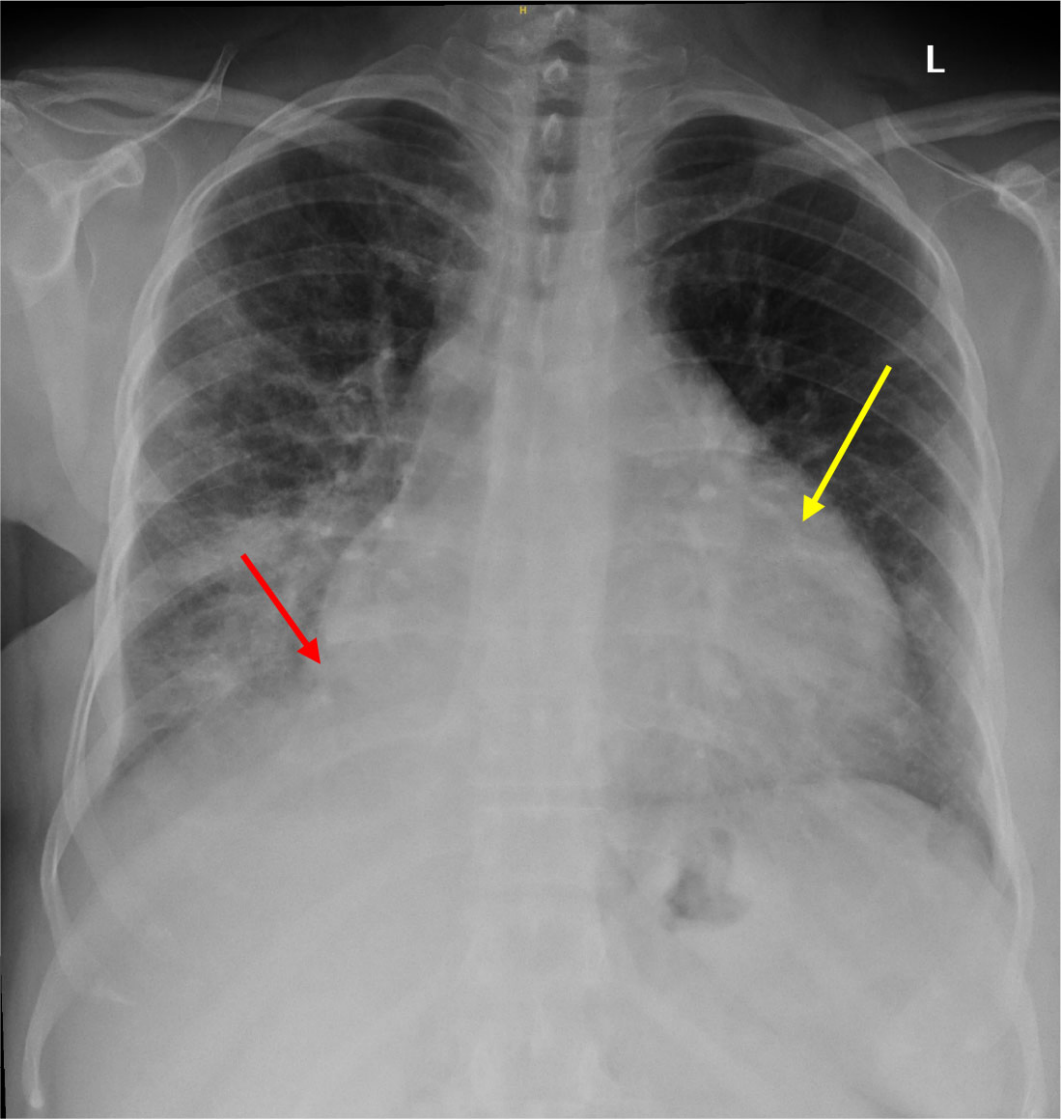
Right lower lobe consolidation with possible pericardial effusion. Chest X-ray demonstrates opacification in the right lower lobe (red arrow), consistent with consolidation. The cardiac silhouette is enlarged (yellow arrow), raising suspicion for a concurrent pericardial effusion.

**Figure 2:**
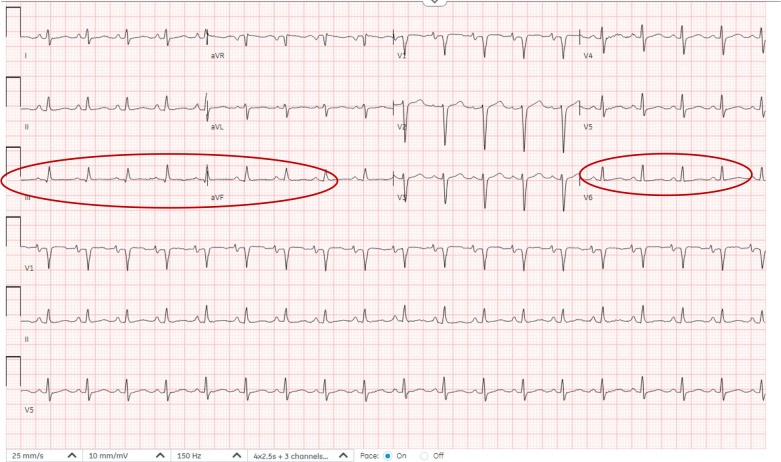
Twelve-lead electrocardiogram (ECG). ECG demonstrating sinus tachycardia at approximately 115 beats per minute with low-voltage QRS complexes and diffuse nonspecific ST-T wave abnormalities, findings consistent with pericardial effusion.

Laboratory tests showed normal hemoglobin (15 g/dL), platelet count (426 x10^9^/L), and WBC (16.9 x10^9^/L). Both electrolyte and renal panels were normal. ESR was 48, CRP 78 mg/L, and procalcitonin level was 0.29 ng/mL. However, the COVID-19 swab was positive, and her chest radiograph showed changes suggestive of possible consolidation in the right lower lobe with an increased cardiac silhouette size, suggesting possible pericardial effusion (**Figure 1**). Her ECG showed sinus tachycardia (**Figure 2**).

**Figure 3:**
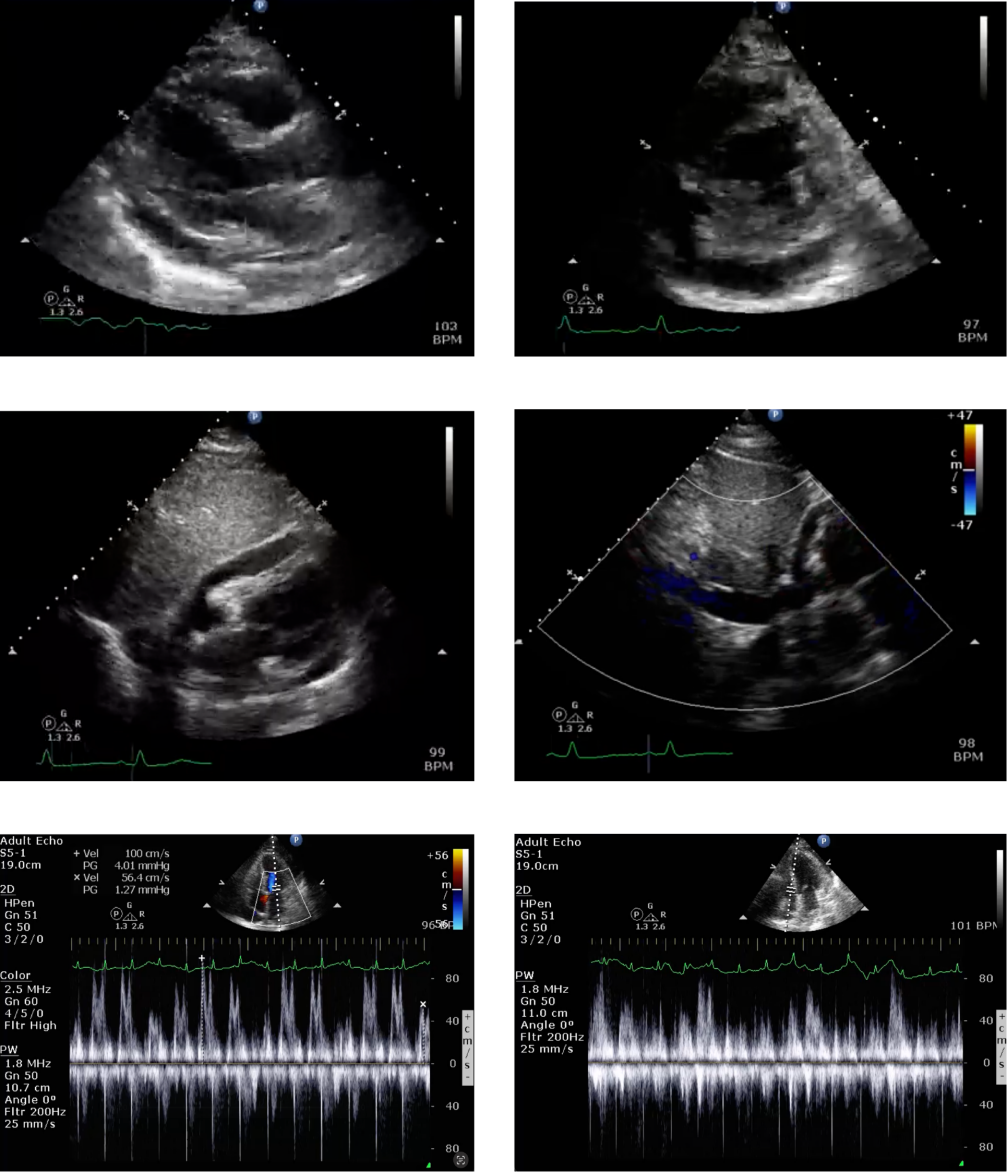
Echocardiographic findings of moderate pericardial effusion with hemodynamic significance. Multiple echocardiographic views demonstrate a moderate-sized pericardial effusion. Key hemodynamic features include diastolic collapse of the right ventricle, systolic inversion of the right atrium, and marked respiratory variation in mitral and tricuspid inflow velocities, consistent with early signs of cardiac tamponade.

She was admitted with COVID-19-related pneumonia and a possible superimposed bacterial infection for which she was treated with antibiotics. The patient’s hospitalization was complicated by progressive tachycardia and tachypnea. Her jugular venous pressure was elevated with a positive pulsus paradoxus, and no evidence of heart failure. Echocardiography revealed a moderate-sized pericardial effusion with signs suggestive of hemodynamic significance, including respiratory variation in mitral and tricuspid flow velocities, diastolic collapse of the right ventricle, and systolic atrial inversion (**Figure 3**).

### 2.3 Management and follow-up

**Figure 4:**
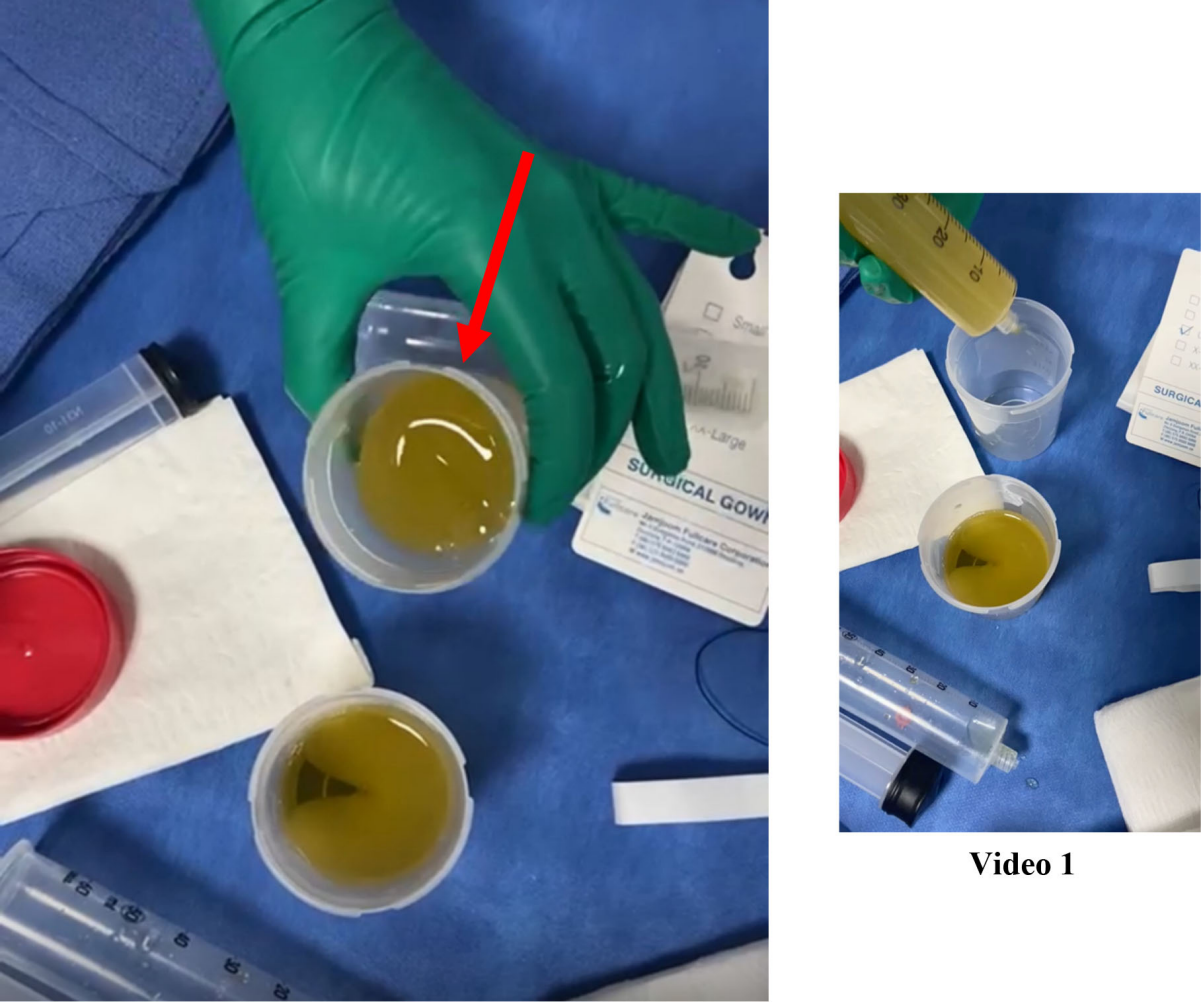
Gross appearance of pericardial effusion. Image showing the aspirated pericardial fluid, which was initially clear and yellow, consistent with a serous effusion. Notably, after standing, the fluid underwent gelatinous transformation (as shown in the attached **Supplementary Video 1**). This sample was obtained during pericardiocentesis for diagnostic and therapeutic purposes.

The patient underwent diagnostic/therapeutic pericardiocentesis. During initial drainage, approximately 500 mL of pericardial fluid was aspirated, which appeared gelatinous. However, following laboratory processing, the fluid was described as clear and serous. However, within a few minutes of drainage, the collected fluid began clotting and reforming into a gelatinous jelly-like material (**Figure 4**, **Supplementary Video 1**). A comprehensive analysis of the pericardial fluid was performed. The key biochemical parameters were as follows: Protein 58.1 gm/L, LDH 250.0 unit/L, Glucose 13.8 mmol/L, pH 7.00, Triglycerides 0.41 mmol/L, and total Cholesterol 2.5 mmol/L. Acid-fast bacilli (AFB) smear and mycobacterial culture were negative. Routine bacterial cultures also showed no growth. Viscosity measurement was not performed, as it is not routinely available in our clinical setting. COVID-19 polymerase chain reaction in the fluid returned positive results, and cytology revealed rare malignant cells, suggesting the possibility of metastatic breast cancer. A few days later, the pericardial effusion recurred and became large in size, requiring another pericardiocentesis. Considering the high risk of pericardial effusion recurrence in the setting of malignancy, she underwent pericardiectomy. The pathology report of the pericardial tissue suggested metastatic breast carcinoma. Metastatic workup revealed bone metastasis, which had been managed by her oncologist since then. At her most recent follow-up, her disease is stable on her current systemic therapy regimen with no reported cardiac consequences, and she reports a good quality of life.

## 3 Discussion

The convergence of cancer and COVID-19 presents significant challenges to healthcare systems worldwide. Both conditions are serious health threats, and their coexistence can have profound implications for patients, healthcare providers, and public health officials. Understanding the interplay between these diseases is crucial for developing effective strategies for their prevention, diagnosis, and treatment (5, 6).

Both conditions may directly or indirectly lead to significant cardiovascular complications, such as cardiomyopathies, acute coronary syndromes, and pericardial diseases such as pericardial effusion and pericardial tamponade. Existing evidence has shown that while both pericardial conditions can occur in patients with breast cancer, they are generally considered rare compared to other metastatic sites such as the lungs, liver, and bones. Generally, patients with metastatic breast cancer exhibit a prevalence of pericardial effusion ranging from 12% to 25%, with a minority developing the life-threatening complication of tamponade (7). This rarity can be attributed to several factors, including the anatomical location of the breast and the nature of breast cancer metastasis. However, the prevalence of pericardial effusion in patients with COVID-19 varies depending on the severity of the infection and the specific population studied. However, multiple studies have shown that pericardial effusion is a relatively common complication, particularly in patients with severe COVID-19, and that its prevalence may vary depending on factors such as geographic location, patient demographics, and healthcare practices, but it is generally reported between 10-15% (8, 9).

The coexistence of COVID-19 and breast cancer may increase the risk of pericardial effusion and tamponade due to a combination of factors, including the metastatic spread of cancer, treatment-related complications of radiation and chemotherapy, direct viral effects, immune-mediated mechanisms, and coagulation abnormalities that may accompany those conditions. While the mechanisms underlying this complication may differ between the two conditions, the potential consequences can be detrimental.

The characteristics of pericardial fluid in malignant pericardial effusions can vary depending on the underlying malignancy and other factors. Generally, it is often hemorrhagic and associated with a positive cytology, similar to malignant pleural effusions (10). Gelatinous or jelly-like consistency and appearance of the fluid were previously reported in malignant pleural effusions in patients with malignancies, such as mesothelioma, melanoma, and some gynecological tumors, and were thought to be secondary to the high concentrations of hyaluronic acid produced by some of these tumors in the pleural fluid (3, 4). Fluid viscosity has been reported as a possible way to differentiate between exudative and transudative pleural effusions. However, to the best of our knowledge, this has never been reported for pericardial effusions.

Analysis of pericardial fluid characteristics in malignant pericardial effusions offers valuable diagnostic insights. Although hemorrhagic effusion and elevated protein content are frequently observed, specific characteristics may vary depending on the underlying malignancy. Cytological examination is crucial for confirming the diagnosis of malignant effusions and identifying the specific malignancies.

Limited data exists regarding the fluid characteristics of pericardial effusions associated with COVID-19. Allam et al. (11) demonstrated that pericardial fluid analysis in a COVID-19 patient exhibited features consistent with exudative effusion, characterized by significant elevations in lactate dehydrogenase and albumin levels. A previous case series indicated that COVID-19-related pleural effusions are predominantly exudative in nature and often exhibit lymphocytic predominance (12).

COVID-19 has profoundly impacted various physiological systems, including hemostasis and blood viscosity. Multiple mechanisms have been proposed to explain the hypercoagulable state observed in COVID-19 patients, including inflammation, endothelial dysfunction and activation, platelet activation, and complement activation. Collectively, these factors contribute to increased plasma viscosity, potentially leading to severe complications.

The gelatinous transformation observed in this case may be explained by a synergistic interaction between malignant pericardial effusion and COVID-19–associated hyperinflammation. Although malignant effusions are well recognized, they are typically serous or hemorrhagic rather than gelatinous, even in the presence of positive cytology. In this patient, malignant cells were identified in the pericardial fluid, providing the cellular basis for the effusion. However, the concurrent positive COVID-19 PCR suggests a superimposed inflammatory process. Severe COVID-19 is characterized by a hyperinflammatory state (cytokine storm) that increases vascular permeability, promoting the extravasation of protein-rich fluid and inflammatory mediators into serous cavities. This proteinaceous exudate, potentially combined with tumor-derived mucins or pro-coagulant factors, may have increased fluid viscosity and contributed to the unusual jelly-like consistency. Thus, while malignancy likely initiated the effusion, the distinctive gelatinous character may reflect the additive effects of COVID-19–induced inflammation and altered coagulative dynamics.

The main strength of this report is its novelty, being the first to describe this phenomenon. However, it has several limitations. As a single case report, its findings are not generalizable. Furthermore, the lack of comprehensive biochemical analysis of the pericardial fluid, including viscosity, protein, and LDH levels, limits our ability to definitively characterize the effusion and pinpoint the exact mechanism behind its gelatinous transformation.

## 4 Patient perspective

The patient was informed about the unusual finding of her pericardial fluid. She was initially alarmed but expressed relief that a diagnosis could be made. She remains under regular follow-up with her oncologist and cardiologist and is grateful that her case might help the medical community understand this rare condition better.

## 5 Conclusion

Pericardial effusion and cardiac tamponade are rare complications in breast cancer. In this case, coexisting COVID-19 may have contributed to the gelatinous nature of the pericardial fluid. To our knowledge, this is the first reported instance. These findings are preliminary, and further studies are needed to confirm their significance and explore potential causal mechanisms.

## 6 Key clinical message

This case highlights the unusual gelatinous appearance of pericardial fluid in malignant effusion with concurrent COVID-19—an observation not previously reported. The underlying pathophysiological mechanisms remain unclear, warranting further investigation to elucidate this phenomenon.

## Data Availability

The original contributions presented in the study are included in the article/**Supplementary Material**. Further inquiries can be directed to the corresponding author.
